# Social Exclusion and Online Aggressive Behavior: Mediation Through Ego Depletion and Moderation Through Mindfulness

**DOI:** 10.3390/bs15030346

**Published:** 2025-03-11

**Authors:** Jing Zhao, Shi-Sheng Chen, Hua Wei, Yu Hu

**Affiliations:** 1 School of Humanities and Social Sciences, North University of China, Taiyuan 030051, China; zj_ccnu@163.com (J.Z.); 13102948673@163.com (Y.H.); 2 Department of Student Affairs, Fujian Polytechnic of Water Conservancy and Electric Power, Sanming 365000, China; chenshisheng@fjsdxy.com; 3 School of Education Science, Qingdao University, Qingdao 266071, China

**Keywords:** online aggressive behavior, social exclusion, ego depletion, mindfulness, college students

## Abstract

The popularity and availability of the Internet has led to a higher frequency of online aggressive behavior, which has aroused increasing attention among researchers. The present study investigated the relationship between social exclusion and online aggressive behavior based on the general aggression model, as well as mediation through ego depletion and moderation through mindfulness. A sample of 953 college students (466 men and 487 women) were recruited to complete questionnaires that assessed social exclusion, online aggressive behavior, ego depletion, and mindfulness. The results showed that social exclusion was significantly and positively associated with online aggressive behavior and that ego depletion played a mediating role in this relationship. Additionally, mindfulness moderated the effect of social exclusion on ego depletion and online aggressive behavior. Specifically, compared to individuals with a high level of mindfulness, individuals with a low level of mindfulness who were experiencing social exclusion were more prone to experiencing ego depletion and partaking in online aggressive behaviors. This study deepens the existing understanding of the mechanisms through which social exclusion affects online aggressive behavior, which could have practical implications for the prevention of and interventions against online aggressive behaviors.

## 1. Introduction

With the rapid development of information technology, the popularity of the Internet has gradually increased. According to the 54th Statistical Report on China’s Internet Development, by the end of June 2024, the total number of Internet users nearly reached 1.1 billion, the Internet penetration rate was 78%, and the average amount of personal time spent online per week was 29 h in China (China Internet Network Information Center [CNNIC], 2024). The Internet has increasingly penetrated every aspect of daily life, including work, study, entertainment, and social interaction. The burgeoning emergence of social media platforms, online forums, and other virtual social arenas has not only diversified the modalities of human social interaction, but has also led to a marked upsurge in the frequency of online social engagements. Concurrently, online aggressive behavior has emerged as an urgent and formidable social quandary. Notably, approximately 60% of college students exhibit online aggressive behavior (T. L. Jin et al., 2018). Therefore, researchers have begun to pay increasing attention to online aggressive behavior as an emerging form of aggression.

The manifestation of aggressive behaviors by the digital generation has transformed traditional bullying dynamics into a more contemporary form (Wong-Lo et al., 2011). Researchers have adopted the term “cyberbullying” to denote aggressive behavior that occurs via digital communication platforms. Smith et al. (2008) defined it as “an aggressive intentional act carried out by a group or individual, using electronic forms of contact, repeatedly and over time against a victim who cannot easily defend him or herself”, which encompasses four core features: intentional harm, the use of technology, an imbalance of power, and repetition. Grigg (2010) examined definition of cyberbullying with thematic analysis and interpretative phenomenological analysis, the result suggested that the term of cyberbullying was inadequate and restricted. Therefore, he proposed a more comprehensive concept, “cyber aggression” (also known as online aggression, online aggressive behavior, digital aggression), and defined it as any behavior carried out with the intention to harm to others through the Internet. This definition does not focus on imbalance of power or repetition of the act. With the emergence of different forms, cyberbullying, online stalking, harassment, stalking, abuse, hostility, and flaming are also included in the category of online aggressive behavior (Grigg, 2010; Mladenovic et al., 2021).

It has been speculated that the increase in online aggressive behavior is fostered by the anonymity facilitated by the Internet, which eliminates important social cues and promotes honest self-expression, free of immediate judgment or consequences (Kurek et al., 2019; Kim et al., 2023). Therefore, online aggressive behavior is time–space free and propagates rapidly, resulting in behavior that is more serious, persistent, and difficult to control than aggressive behavior in real life (Z. E. Ding et al., 2018). Previous studies have shown that the experience of online aggression is suggestive of depression, anxiety, and even suicide (Arnon et al., 2022; Molero et al., 2022; Silva et al., 2017). A meta-analysis of 12 studies found that the victims of online aggressive behavior by their peers exhibited a higher level of school attendance and academic achievement difficulties (Gardella et al., 2017). And individuals who have endured online aggressive behaviors are more likely to engage in substance abuse, rule breaking, or delinquent behavior (Crane et al., 2021; Farrell et al., 2020). In short, online aggressive behaviors are considered to have a series of negative impacts on the victims. However, is that all? No, of course not. Perpetrators of online aggressive behavior do not immediately “see” the impact of their behavior on others and even distort the consequences of their action. Therefore, they may perceive that online aggressive behavior is encouraged, which can promote its generalization into other areas and situations (Yahner et al., 2015), leading to more serious consequences, such as legal consequences (Paul et al., 2012). Therefore, online aggressive behaviors also cause harm to the aggressors. In addition, social contagion effects are observed in regard to aggressive behavior, and the Internet amplifies this effect, where the spread can be faster and involve more people (Trifiletti et al., 2021), such as online firestorms. This phenomenon not only destroys the harmonious environment of the Intenet, but may also cause serious social contradictions, subverting social stability. Therefore, based on the above perspectives of victims, aggressors, and society, online aggressive behavior has emerged as a phenomenon that can not be neglected and demands an immediate resolution, so delving deeply into the causes, underlying mechanisms, and, subsequently, formulating effective countermeasures have become imperative.

From the perspective of different disciplines, researchers have explored factors influencing online aggressive behavior. Psychologists mainly focus on personal profiles, such as dark triad personality (Kurek et al., 2019), narcissism (Y. Y. Chen et al., 2022), and emotion regulation strategies (Quintana-Orts et al., 2023). Educationalists and sociologists pay more attention to external social forces, such as harsh parenting (J. L. Wu, 2022) and group norms (Trifiletti et al., 2021). While communication scientists are more concerned with the network environment, including aspects such as anonymity (Y. Y. Chen et al., 2022) and exposure to violence (Santos et al., 2023). However, these research results were based on a single perspective or hypothesis. The general aggression model is considered to be a relatively extensive and comprehensive theoretical framework, which is used to elucidate cyberbullying (Allen et al., 2018; Kokkinos & Antoniadou, 2019).

The general aggression model is separated into proximate and distal processes (Allen et al., 2018). The distal processes focus on biological and persistent environmental factors, while the proximate processes contain three stages of input, routes, and outcomes. In the present study, we mainly explore the proximate processes of online aggressive behavior. In regard to the proximate processes, person-related and situational factors in the stage of inputs act additively or interactively on the routes, which influence the appraisal and decisions in terms of the outcomes. Boman and Mowen (2019) suggested that the influence of interpersonal factors on social adaptation is prominent among college students, and social exclusion is a common negative interpersonal experience. However, only a few researches have focused on the relationship between social exclusion and online aggressive behavior, so we decided we would try to explore it. Person-related factors, which are relatively stable over time and across situations, may influence how a person responds to a situation (Allen et al., 2018). In recent years, mindfulness as a protective factor for individuals has received extensive attention. A published literature review on mindfulness and social exclusion suggests that mindfulness can effectively mitigate the negative effects of social exclusion and promote recovery from social exclusion (J. Chen et al., 2022). Above all, based on the general aggression model, the present study will examine the influence of social exclusion on online aggressive behavior, and will assess whether mindfulness plays a buffering role in this relationship, in order to provide constructive suggestions for the prevention of and interventions targeting online aggressive behavior.

### 1.1. Social Exclusion and Online Aggressive Behavior

Social exclusion (also termed ostracism, rejection) is a phenomenon wherein people are rejected or repelled by another person or group (Du & Xia, 2008). According to the temporal need–threat model (Williams, 2009), social exclusion refers to the destruction of social ties, which threatens the fundamental psychological needs related to belonging and self-esteem and leads to social pain that is comparable to physical pain. Thus, social exclusion is considered to be a frustrating experience, which is to be strenuously avoided (Shao & Zhang, 2021). According to the frustration–aggression hypothesis, aggression is a hostile response to frustration, so the excluded person adopts reactive aggressive behavior for the purpose of revenge (Dollard & Miller, 1939). From the perspective of the general aggression model, social exclusion is an important and common frustrating situation that destroys individuals’ internal state (cognition, emotion, and arousal) and leads to aggressive behavior (Anderson & Bushman, 2002). Quan et al. (2024) used questionnaires to examine the relationship between long-term social exclusion and aggression, and conducted an experiment using the Cyberball paradigm to examine whether short-term social exclusion influences aggressive behavior; the results suggested that both long-term and short-term social exclusion were associated with aggressive behavior, and that hostile attribution played a mediating role. A meta-analysis of 92 studies found that social exclusion has a significant positive correlation with aggression (J. J. Jin et al., 2023).

With the popularity of the Internet, various online platforms have emerged to provide space for aggressive behavior. The cue-filtering theory points out that individuals can hide some or even all of their true identity online, which not only leads to the loss of a sense of presence, but also hinders the transmission of social cues, as a result, individuals may exhibit more extreme behaviors (Joinson, 2003). Studies have shown that individuals who engaged in offline social exclusion or bullying turn into perpetrators of online aggressive behavior in order to seek a psychological balance and reduce the risk of retaliation in reality (Liang et al., 2023; Paez & Hart, 2021; Zhao et al., 2024). And a piece of cross-lag research showed that there was a long-term effect of social exclusion on online aggressive behavior (Sun et al., 2024). Therefore, we proposed the following hypothesis:

**H1.**  *Social exclusion is positively correlated with online aggressive behavior.*

### 1.2. Ego Depletion as a Mediator

According to the general aggression model, an excluded individual will trigger negative cognition, affect, and arousal in the route stage, which involves the consumption of psychological resources. In terms of the cognition route, the experience of social exclusion will trigger individuals’ hostile state (such as hostile automatic thoughts, hostile attribution bias) and rumination, while suppressing these thoughts requires more cognitive resources (Quan et al., 2024; Sun et al., 2024; H. Wang et al., 2024). Social exclusion leads to increased negative emotions (such as anger, loneliness), which require more resources for emotional regulation (Brinker et al., 2023; He et al., 2022; Leary, 2015). Physiological studies have also demonstrated that excluded individuals exhibit an increase in cortisol levels and activation of the left anterior cingulate cortex (ACC), which indicate that social exclusion triggers individuals’ stress response and shares similar neural mechanisms as physical pain (Dickerson & Kemeny, 2004; Eisenberger et al., 2003). However, a person’s mental resources are limited, and Baumeister et al. (1998) proposed the concept of “ego depletion”, which refers to a state in which individuals have less cognitive resources after self-regulation activities (Goto & Kusumi, 2013). Baumeister et al. (2024) pointed to interpersonal conflict as a major cause of ego depletion. Y. Zhang et al. (2016) reported that performance (accuracy) in regard to cognitive reasoning and Stroop tasks was poorer in a social exclusion group than in a control group, indicating a significant main effect of social exclusion on ego depletion. Another study indicated that workplace ostracism is significantly negatively correlated with ego depletion (Zhu et al., 2020).

The third stage of the general aggression model focuses on appraisal and decision-making processes. If a person does not have sufficient time and mental resources, immediate negative appraisals of social exclusion are more likely to be directly enacted as aggressive behaviors (Allen et al., 2018; Shao & Zhang, 2021). Previous research has shown that when ego depletion occurs individuals tend to be aggressive (Barlett et al., 2016; X. X. Wang et al., 2021). Online aggressive behavior is a common form of aggression that allows individuals to vent their anger and dissatisfaction through the Internet. One study found that ego depletion is significantly and positively correlated with online aggressive behavior (Q. Ding et al., 2020). Hence, we formulated the following hypothesis:

**H2.**  *Ego depletion mediates the relationship between social exclusion and online aggressive behavior.*

### 1.3. Mindfulness as a Moderator

Mindfulness has its roots in Buddhism. Mindfulness-based stress reduction (MBSR) was applied in regard to patients’ pain management and stress management at the University of Massachusetts Medical Center in 1979, in which mindfulness was divorced from religion (Kabat-Zinn, 2003). Ever since, an extensive array of research and practical applications have been conducted centered around mindfulness, propelling its gradual acceptance within mainstream society. It is defined as the capacity for receptive, non-judgmental, conscious, and open attention to a person’s own thoughts, emotions, and physical states occurring at the present moment (Brown & Ryan, 2003).

According to the re-perceiving model of mindfulness, mindfulness enables individuals to intentionally observe moment-by-moment experiences with openness and non-judgment, eliminate automatic or reactive behavioral and emotional patterns, and foster more adaptive coping skills in response to negative stimulation (Shapiro et al., 2006). The mindfulness coping model points out that positive cognitive reassessment can effectively alleviate the negative effects of stressful events, in which mindfulness plays a central role in that positive cognitive reassessment (Garland et al., 2009). Previous research indicates that mindfulness helps individuals to alleviate problematic behaviors caused by social exclusion. For example, Arslan and Coşkun (2022) discovered a detrimental effect of social exclusion on Internet addiction, with self-forgiveness mediating the relationship and mindfulness moderating the mediating role of self-forgiveness. Additionally, mindfulness is negatively associated with aggressiveness and cyberbullying (Xiong et al., 2023; Yu et al., 2022). Furthermore, Gillions et al.’s (2019) systematic review of 22 studies showed that mindfulness-based interventions effectively reduced aggression and violence in adults. Accordingly, a third hypothesis was proposed:

**H3.**  *Mindfulness plays a moderating role between social exclusion and online aggressive behavior.*

Mindfulness can help individuals to separate their self-concept from specific events (Yuan et al., 2024); thus, individuals with a high level of mindfulness can separate themselves from the negative emotions caused by social exclusion, minimize threats to their self-worth, and decrease automatic cognition, and thus reduce the consumption of cognitive resources. According to the conservation of resources theory, individuals with greater resources are less vulnerable to resource loss, whereas those who lack resources are more likely to experience resource loss (Hobfoll et al., 2018). Mindfulness can be regarded as a valuable resource, with two core connotations of ‘control’ and ‘resilience’ (Liu & Sun, 2022). Thus, when individuals with a high level of mindfulness experience social exclusion, they are less likely to experience significant resource loss. Furthermore, previous studies have shown that mindfulness moderates the effect of daily illegitimate tasks on ego depletion (Yuan et al., 2024), and that mindfulness-based training interventions can reduce individuals’ ego depletion in the workplace (X. Zhang et al., 2022). Accordingly, the current research tested the following hypothesis:

**H4.**  *Mindfulness moderates the relationship between social exclusion and ego depletion.*

### 1.4. The Present Study

**Figure 1:**
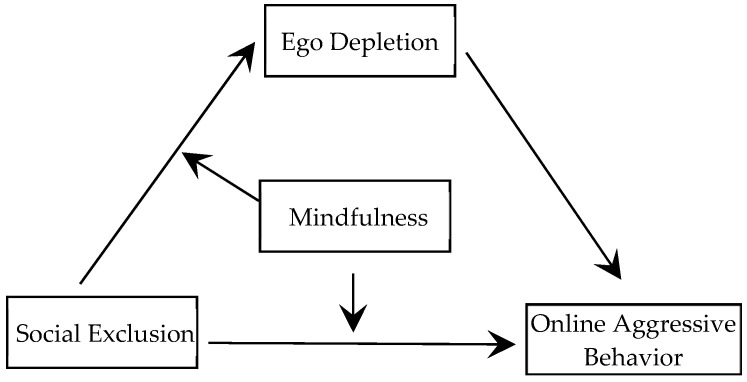
Proposed moderated mediation model.

The present study examines the relationship between social exclusion and online aggressive behaviors, based on the general aggression model. It also explores the mediating effect of self-depletion and the moderating effect of mindfulness. The proposed integrated model is illustrated in Figure 1.

## 2. Materials and Methods

### 2.1. Participants

Convenience sampling was adopted to recruit college students from universities located in Shanxi Province and Fujian Province. After obtaining informed consent, 1050 participants anonymously completed a questionnaire that took approximately 10 min to complete and 97 participants who reported the same values for all the items were excluded. The remaining 953 valid responses were analyzed. Among the total sample (M_age_ = 20.20 years, SD_age_ = 1.51, age range = 17–24 years), 466 and 487 participants were men and women, respectively; 483 were undergraduate students and 470 were vocational college students.

### 2.2. Measurements

Online aggressive behavior. To assess online aggressive behavior, we adapted the online framing subscale from the Scale of Adolescent Internet Deviance compiled by X. H. Ma and Lei (2010). The scale comprises 20 items, such as ‘when I have a conflict with someone online, I will send them offensive symbols/pictures’. All the items are rated using a five-point Likert scale (1 = never, 5 = always). Higher scores indicate greater engagement in online aggressive behavior. In this study, the fit indexes of the confirmatory factor analysis were as follows: χ^2^/df = 3.65, NFI = 0.87, GFI = 0.89, and CFI = 0.90. The Cronbach’s alpha value for the scale was 0.89.

Social exclusion. The Social Exclusion Questionnaire for Undergraduates developed by H. J. Wu et al. (2013) was adopted. The scale can be divided into two dimensions: direct exclusion (e.g., ‘I’ll be picked on maliciously’) and indirect exclusion (e.g., ‘When I am down, no one can give me advice or comfort’). The scale comprises 19 items, rated from 0 (rarely) to 4 (always) (H. J. Wu et al., 2013). Higher scores indicate a greater frequency of social exclusion. The fit indexes of the confirmatory factor analysis were as follows: χ^2^/df = 2.15, NFI = 0.89, GFI = 0.92, and CFI = 0.90. Cronbach’s alpha was 0.89 in the present study.

Ego depletion. The simplified Ego Depletion Scale, with five items (e.g., ‘I feel exhausted’), was used to assess ego depletion (Lanaj et al., 2014). This scale has been translated and used in Chinese studies, with adequate validity and reliability. Items are scored using a seven-point scale, ranging from 1 (strongly disagree) to 7 (strongly agree). A higher score indicates a higher level of ego depletion. In the current study, confirmatory factor analysis of the scale was carried out, and the fit indexes were as follows: χ^2^/df = 4.47, NFI =0.96, GFI = 0.92, and CFI = 0.96. Cronbach’s α was 0.86.

Mindfulness. The Mindful Attention Awareness Scale (MAAS), developed by Brown and Ryan (2003) and revised by S. Y. Chen et al. (2012), was used to assess mindfulness. The scale includes 15 items, such as ‘I rush through activities without being really attentive to them’. The items are rated using a six-point Likert scale, ranging from 1 (almost always) to 6 (almost never). A higher score indicates high trait mindfulness. Confirmatory factor analysis of the scale was carried out, and the fit indexes were as follows: χ^2^/df = 3.47, NFI = 0.92, GFI = 0.87, and CFI = 0.92. Cronbach’s alpha was 0.87 in the present study.

### 2.3. Data Analysis

All the data were analyzed using SPSS 25.0. First, descriptive statistics and correlations were obtained. Second, the PROCESS macro for SPSS was used to test the moderated mediation model using 5000 bias-corrected samples. The effect was considered significant when the 95% confidence interval (CI) did not include zero (Hayes, 2013). In particular, model 4 was used to test the mediating model with ego depletion, and model 8 was used to test the integrated model with ego depletion as the mediator and mindfulness as the moderator.

## 3. Results

### 3.1. Preliminary Analysis

Table 1 presents the mean, standard deviation, and Pearson’s correlations for the main variables. Social exclusion was positively correlated with ego depletion and online aggressive behavior, ego depletion was positively correlated with online aggressive behavior, and mindfulness was negatively correlated with social exclusion, ego depletion, and online aggressive behavior.

In addition, multicollinearity diagnostics were performed using SPSS 25.0 through the adoption of linear regression procedures, with the Variance Inflation Factor (VIF) thresholds set at 5 (warning level) and 10 (critical level), following contemporary guidelines (O’Brien, 2007). The predictor variables were standardized (z-score transformation) prior to the analysis to reduce non-essential collinearity. The VIF values ranged from 1.78 to 4.29 (VIF_social exclusion_ = 3.03; VIF_ego depletion_ = 4.29; VIF_mindfulness_ = 1.78), and all values remained below the warning level, suggesting that there was no severe multicollinearity.

### 3.2. Mediating Role of Ego Depletion

Model 4 in regard to the SPSS macro PROCESS (Hayes, 2013) was chosen to examine the potential association between social exclusion and online aggressive behavior, as well as the possible mediating role of ego depletion. Previous studies have shown that gender, age, and time spent online are related to online aggressive behavior (Barlett & Coyne, 2014; Favini et al., 2024; Guidi et al., 2022; T. L. Jin et al., 2024); therefore, we included them as covariates in all the analyses.

As shown in Table 2, social exclusion positively predicted online aggressive behavior, social exclusion positively predicted ego depletion, and ego depletion positively predicted online aggressive behavior. In other words, ego depletion partially mediated the association between social exclusion and online aggressive behavior. The total effect of social exclusion on online aggressive behavior was 0.78 (Bootstrap SE = 0.02; Bootstrap 95% CI = [0.74, 0.82]), the direct effect was 0.40 (Bootstrap SE = 0.03; Bootstrap 95% CI = [0.34, 0.46]). The completely standardized indirect effect of social exclusion on online aggressive behavior was 0.38 (Bootstrap SE = 0.04; Bootstrap 95% CI = [0.31, 0.44]), and the ratio of the indirect to the total effect was 48.47% (Bootstrap SE = 0.05; Bootstrap 95% CI = [0.66, 1.37]).

### 3.3. Moderated Mediation

Model 8 in regard to the SPSS macro PROCESS (Hayes, 2013) was constructed to investigate whether mindfulness moderated the association between social exclusion, ego depletion, and online aggressive behavior. The results of the mindfulness moderation test are presented in Table 3. The main results comprise two parts, the regression analysis model and conditional effect analysis, which are presented in Table 3 and Table 4, respectively.

**Figure 2:**
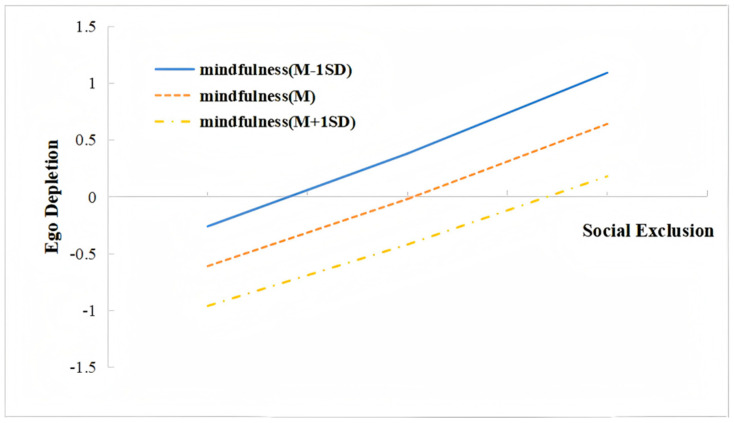
Interaction effect of social exclusion and mindfulness on ego depletion.

As shown in model 1 in Table 3, the interaction of social exclusion and mindfulness had a significant predictive effect on ego depletion (β = −0.05, t = −3.29, *p* < 0.01), indicating that mindfulness played a moderating role in the association between social exclusion and ego depletion. For descriptive purposes, we plotted predicted social exclusion against ego depletion, separately for three levels [at the mean of mindfulness, as well as one standard deviation above and below the mean of mindfulness (M, M ± 1SD)] (Figure 2). Simple Slop tests showed that for college students with a high level of mindfulness, social exclusion significantly predicted ego depletion (B = 0.60, t = 23.44, *p* < 0.001), the predictive effects were stronger for individuals with medium levels of mindfulness (B = 0.65, t = 38.20, *p* < 0.001), and the effects were even stronger for individuals with low levels of mindfulness (B = 0.71, t = 32.62, *p* < 0.001). The impact of the R^2^ change in the interaction between social exclusion and mindfulness on ego depletion was 0.02 (F = 10.83, *p* <0.01), and Cohen’s f^2^ was 0.11, indicating a small effect size (Cohen, 1988).

**Figure 3:**
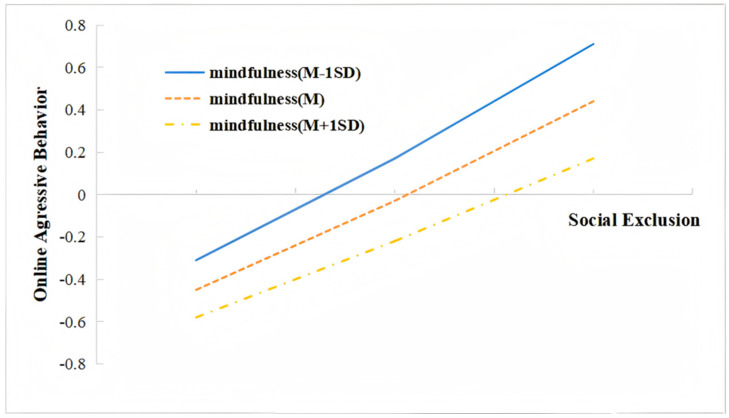
Interaction effect of social exclusion and mindfulness on online aggressive behavior.

Moreover, model 2 in Table 3 shows that the interaction between social exclusion and mindfulness had a significant predictive effect in regard to online aggressive behavior (β = −0.07, t = −3.82, *p* < 0.01). We plotted predicted social exclusion against online aggressive behavior separately for three levels (M, M ± 1SD) (Figure 3). Simple Slop tests showed that social exclusion significantly predicted online aggressive behavior in individuals with a high level of mindfulness (Bsimple = 0.40, t = 11.16, *p* < 0.001), the predictive effects were stronger for individuals with medium levels of mindfulness (Bsimple = 0.47, t = 15.47, *p* < 0.001), and the effects were even stronger for individuals with low levels of mindfulness (Bsimple = 0.5, t = 15.34, *p* < 0.001) (seen as the “direct effect” in Table 4). The impact of the R^2^ change in the interaction between social exclusion and mindfulness on ego depletion was 0.04 (F = 14.64, *p* < 0.001), and Cohen’s f^2^ was 0.15, indicating a medium effect size (Cohen, 1988).

As shown in Table 4, the values of the indirect effects varied slightly at three levels of mindfulness, and the index of the moderated mediation was −0.02 (Bootstrap SE = 0.01; Bootstrap 95% CI = [−0.04, 0.004]), indicating that the indirect effects of social exclusion on online aggressive behavior via ego depletion were not significantly moderated by mindfulness.

## 4. Discussion

Based on the general aggression model, this study examined the association between social exclusion and online aggressive behaviors, and the underlying mechanisms of ego depletion and mindfulness.

### 4.1. Social Exclusion and Online Aggressive Behavior

This study affirmed that social exclusion was significantly and positively associated with college students’ online aggressive behavior, which is consistent with previous research (Zhao et al., 2024; Sun et al., 2024). Thus, H1 is confirmed. The result validates the perspective of the frustration–aggression hypothesis. The social connections of the excluded individual become fragmented, posing a serious threat to their basic requirements for a sense of belonging and in terms of their self-esteem. This situation causes the individual to endure social pain and results in them resorting to aggressive behaviors as a means of alleviating such distress. According to the cue-filtering theory, the lack of social cues significantly bolsters the anonymity inherent in the online environment. Given the convenience and anonymity of the Internet, online aggressive behavior is more subtle and less costly compared with face-to-face aggression (Niu et al., 2015); thus, it is more likely to become a new approach for those who are excluded from real life interactions to vent their frustrations. In addition, excluded individuals are at a disadvantage in real life, as it is difficult for them to directly retaliate against those who have excluded them. However, the Internet largely nullifies the power advantages in reality and provides a possibility for excluded individuals to engage in online aggressive behavior (Liang et al., 2023). This study further expands the applicability of the general aggression model to the online context. It validates the crucial role that situational factors, such as social exclusion, play in precipitating online aggressive behavior.

### 4.2. Ego Depletion as a Mediator

The results demonstrated that ego depletion acted as a mediator between social exclusion and online aggressive behavior, which supports H2. From one perspective, social exclusion significantly positively predicted ego depletion, consistent with the results in a previous study (Y. Zhang et al., 2016). Social exclusion can lead to adverse cognitive outcomes (including heightened hostility and rumination), evoke negative emotions (such as anger, loneliness), and result in elevated physiological arousal (Brinker et al., 2023; Dickerson & Kemeny, 2004; He et al., 2022; Sun et al., 2024; H. Wang et al., 2024; Williams & Nida, 2011). In such circumstances, individuals frequently need to regulate their negative cognition, emotions, and arousal. However, such regulation consumes their limited self-control resources (DeWall et al., 2011; Mani et al., 2013). In other words, grappling with the social exclusion-associated negative emotions and cognition depletes the individual’s finite mental energy, specifically self-control resources, causing them to fall into a state of ego depletion. This result provides empirical evidence in support of the route stage of the general aggression model, that is, social exclusion has an impact on an individual’s internal state.

On the other hand, ego depletion significantly and positively predicted online aggressive behavior, which is also consistent with the results in previous research (Q. Ding et al., 2020). When individuals’ resources are exhausted, they lose their willingness and ability to exert self-control (Stenseng et al., 2015); thus, they are unable to weigh conflicts rationally and tend to make irrational decisions, such as engaging in aggression. This process is known as the execution mechanism of ego depletion, and it impedes rational decision making (Y. Ma et al., 2020). Situational characteristics (such as social norms and decision-related costs) are critical boundaries that affect execution mechanisms. Compared with offline aggression, the social norms of online aggressive behavior are relatively vague, and the decision cost is lower, which makes depleted individuals more inclined to engage in online aggressive behavior. Finally, the results further confirmed the third stage of the general aggression model, namely that when their resources are insufficient, excluded individuals are inclined to be aggressive based on an immediate appraisal of social exclusion (Allen et al., 2018; Shao & Zhang, 2021). In summary, social exclusion exerts an influence on online aggressive behavior via ego depletion.

### 4.3. Mindfulness as a Moderator

The results showed that mindfulness played a moderating role in terms of the direct effects of social exclusion on online aggressive behavior, which validates H3. Specifically, compared with individuals with a high level of mindfulness, those who with low mindfulness are more likely to engage in online aggressive behavior when excluded. According to the re-perceiving model of mindfulness, when individuals with high levels of mindfulness are excluded, they are more inclined to shift their attention from social exclusion to the present moment. This allows individuals to scrutinize the whole from a more comprehensive perspective and prevents them from falling into the victim mentality, which improves the flexibility of their cognition and emotions, thereby reducing online aggressive behavior (Shapiro et al., 2006). In addition, monitor and acceptance theory, a new mindfulness theory, pinpoints two pivotal components: monitoring and acceptance (Y. Y. Zhang et al., 2022). Monitoring skills are capable of heightening the awareness of individuals’ present moment; meanwhile, acceptance is an emotional regulation strategy that modifies the way individuals interact with their immediate experiences and reduces their emotional reactivity. Those with a high level of mindfulness who endure social exclusion are likely to boost their awareness via monitoring and acceptance. This, in turn, can effectively elevate the level of their cognitive functions and curtail negative reactions, such as online aggressive behavior.

The results suggest that mindfulness played a moderating role between social exclusion and ego depletion, supporting H4. In other words, when faced with social exclusion, individuals with a low level of mindfulness experience a greater degree of ego depletion than those with a high level of mindfulness. According to the conservation of resources theory, individuals strive to obtain, retain, and foster effective psychological resources, and enhanced resources are conducive to coping with stress (Hobfoll et al., 2018). Consequently, individuals with high levels of mindfulness have rich resources to help them cope with the stress of social exclusion, and exhibit a lower level of ego depletion. Although mindfulness moderated the first path in terms of the mediating effect, its moderating effect on the entire mediating effect was not significant. The underlying cause is that mindfulness fails to moderate the effect of ego depletion on online aggressive behavior. To elaborate, regardless of the level of an individual’s mindfulness, when in a state of ego depletion, the individual has great difficulty in restraining impulsive behaviors and is more inclined to engage in online aggressive behavior.

In short, the results confirmed the input stage of the general aggression model, namely that person-related and situational factors can work additively or interactively to influence individuals’ online aggressive behavior (Allen et al., 2018).

## 5. Implications and Limitations

This study explored the mediation and moderation mechanisms between social exclusion and online aggressive behavior, providing valuable insights. Theoretically, this study expands the application of the general aggression model to online contexts, providing a theoretical basis for subsequent research on online aggressive behavior. Practically, the results may inform methods of preventing and intervening to combat online aggressive behavior. First, based on the direct and mediating effects of social exclusion on online aggressive behaviors, mental health educators should pay more attention to excluded individuals, take measures to improve their social skills, and reduce the possibility of social exclusion. Second, ego depletion plays a mediating role between social exclusion and online aggressive behavior; thus, it is necessary to implement interventions to reduce or recover from ego depletion as quickly as possible. One approach could be to improve individuals’ resistance to ego depletion, such as by cultivating the ability to meditate and block out distractions, and increase their initial resources (Hobfoll et al., 2018; Y. Ma et al., 2020). Another approach could be to improve individuals’ ability to recover from ego depletion, such as by viewing a restorative environment and engaging in episodic future thinking (Y. Ma et al., 2020). Third, mindfulness is a buffer that mitigates the impact of social exclusion against ego depletion and online aggressive behavior; thus, mental health educators should provide mindfulness-based interventions.

Despite the valuable insights provided by this study, certain limitations should be acknowledged. First, the cross-sectional design has limitations in regard to establishing causality. Although our results are consistent with the possibility that excluded people consume more psychological resources and are thus more likely to initiate online aggressive behaviors, further experimental and longitudinal studies are essential to establish more definitive causal relationships. Second, the samples in this study are exclusively from Chinese college students, leading to a limitation in terms of result generalizability. The students share similarities in terms of their age, educational background, and social experiences. As a result, the applicability of the research results may be restricted to this specific group. According to the general aggression model, persistent environmental factors are a crucial element in distal processes, such as cultural norms. Previous research has shown that individualism was associated with a higher incidence of online aggressive behavior, whereas collectivism was linked to a lower prevalence of such behavior (Wright et al., 2015). In addition, age also affects online aggressive behavior, females exhibit more online aggressive behavior than males during early adolescence, while males display more such behavior in later adolescence (Barlett & Coyne, 2014). Therefore, future studies should use more diverse and even cross-cultural samples to improve the generalizability of the results.

## 6. Conclusions

This study provides evidence that social exclusion was positively associated with online aggressive behavior. Furthermore, ego depletion partially mediated this relationship. Finally, mindfulness acted as a buffering factor, mitigating the impact of social exclusion on online aggressive behavior and ego depletion. This conclusion verifies the applicability of the general aggression model in the network scenario, and provides effective measures for the prevention of and interventions targeting online aggressive behavior.

## Figures and Tables

**Table 1:** Mean, standard deviation, and correlation coefficients of the main variables.

Variables	M (SD)	1	2	3	4
Social Exclusion	0.69 (0.76)	1			
Ego Depletion	2.46 (1.19)	0.80 **	1		
Mindfulness	3.97 (1.27)	−0.36 **	−0.62 **	1	
Online Aggressive Behavior	1.82 (0.73)	0.79 **	0.80 **	−0.54 **	1

Note: ** *p* < 0.01.

**Table 2:** Mediation analysis.

Dependent Variable	Independent Variables	R^2^	F	β	Bootstrap LLCI	Bootstrap ULCI	t
Ego Depletion	Social Exclusion	0.64	418.61 ***	0.80	0.76	0.84	40.17 ***
(model 1)	Gender			−0.03	−0.10	0.05	−0.66
	Age			0.00	−0.03	0.03	0.003
	Time Spent Online Per Day			0.001	-0.02	0.02	0.10
Online Aggressive Behavior (model 2)	Social Exclusion	0.70	440.58 ***	0.40	0.34	0.46	13.41 ***
	Ego Depletion			0.47	0.41	0.53	15.90 ***
	Gender			0.08	0.01	0.15	2.29 *
	Age			-0.02	-0.04	0.002	−1.78
	Time Spent Online Per Day			0.001	-0.01	0.01	0.11

Note: * *p* < 0.05, *** *p* < 0.001; LL = lower limit, UL = upper limit, CI = confidence interval.

**Table 3:** Moderated mediation analyses.

Dependent Variable	Independent Variables	R^2^	F	β	Bootstrap LLCI	Bootstrap ULCI	t
Ego Depletion	Social Exclusion	0.77	528.63 ***	0.66	0.62	0.69	38.20 ***
(model 1)	Mindfulness			−0.40	−0.43	−0.36	−23.18 ***
	Social Exclusion × Mindfulness			−0.05	−0.09	−0.02	−3.29 **
	Gender			−0.05	−0.11	0.01	−1.54
	Age			0.003	−0.02	0.02	0.25
	Time Spent Online Per Day			−0.04	−0.02	0.01	−0.69
Online Aggressive Behavior	Social Exclusion	0.72	348.58 ***	0.47	0.41	0.53	15.47 ***
(Model 2)	Ego Depletion			0.29	0.22	0.36	8.20 **
	Mindfulness			−0.20	−0.24	−0.15	−8.34 ***
	Social Exclusion × Mindfulness			−0.07	−0.11	−0.03	−3.82 ***
	Gender			0.06	−0.004	0.13	1.85
	Age			−0.02	−0.04	0.003	−1.70
	Time Spent Online Per Day			−0.002	−0.02	0.01	−0.29

Note: ** *p* < 0.01, *** *p* < 0.001.

**Table 4:** Conditional direct and indirect effect analysis.

Level of Mindfulness	Conditional Effect	Effect Value	Bootstrap SE	Bootstrap LLCI	Bootstrap ULCI
Direct Effect	M − 1 SD	0.54	0.04	0.47	0.60
	M	0.47	0.03	0.41	0.53
	M + 1 SD	0.40	0.04	0.33	0.47
Indirect Effect	M − 1 SD	0.21	0.04	0.13	0.30
	M	0.19	0.04	0.12	0.27
	M + 1 SD	0.18	0.04	0.11	0.24

## Data Availability

The data from this study are available from the corresponding author upon reasonable request.
